# *Pediatric Discovery*: Opportunities and challenges in pediatric medicine

**DOI:** 10.1002/pdi3.4

**Published:** 2023-05-30

**Authors:** Qiu Li, Yaolong Chen, Tong‐Chuan He

**Affiliations:** ^1^ The National Clinical Research Center for Child Health and Disorders of China Chongqing China; ^2^ Department of Nephrology Children's Hospital of Chongqing Medical University Chongqing China; ^3^ Chinese GRADE Center WHO Collaborating Centre for Guideline Implementation and Knowledge Translation Lanzhou China; ^4^ Institute of Health Data Science Lanzhou University Lanzhou China; ^5^ Molecular Oncology Laboratory Department of Orthopaedic Surgery and Rehabilitation Medicine The University of Chicago Medical Center Chicago Illinois USA

We are excited about launching the new open access journal, *Pediatric Discovery*, which aims to promote and advance the health and well‐being of infants, children, and adolescents by disseminating cutting‐edge discoveries and knowledge about all aspects of pediatric medicine. This new journal will serve as an essential platform for illuminating basic, translational, and clinical discoveries that impact pediatric health and development.

Pediatrics, a clinical medicine specialty, focuses on the medical care and health management of infants, children, and adolescents from birth up to the age of 18.^1^ However, the American Academy of Pediatrics recommended people seek pediatric care through the age of 21 in the 1960s and eliminated the upper age limit for pediatric care in 2017,^2^ based on the rationale that the transition to adult care should be specific to patient needs, not an arbitrary number. The term pediatrics was first introduced in English in 1859 by a German physician, Dr. Abraham Jacobi (1830–1919), who became the first dedicated pediatrician in the world and is known as the father of American pediatrics. Nonetheless, child‐specific medical problems, such as childhood epilepsy, rashes, premature births, and meningitis, were described in the *Hippocratic Corpus* in the fifth century B.C., as well as by Greek philosophers and physicians Celsus, Soranus of Ephesus, Aretaeus, Galen, and Oribasius from the first to fourth centuries A.D.^3^ Some of the early publications specifically focusing on children's medical concerns appeared between the 1790s and the 1920s.

Pediatrics has now emerged as a major branch of medical science and encompasses a broad spectrum of physical, mental, and social health care, ranging from preventive health care to the diagnosis and treatment of acute and chronic diseases. The field has been evolving at an unprecedented pace due to the advances in our understanding of human diseases at the genome, cell and organ levels. The overall goals of pediatric medicine are to reduce the rates of infant and child deaths, control the spread of infectious diseases, promote healthy lifestyles for a long disease‐free life, and to help improve the lives of children and adolescents with chronic conditions. Pediatric practitioners are also involved in the prevention, early diagnosis, and management of developmental delays and disorders, behavioral problems, functional disabilities, social stresses, and mental disorders, including depression and anxiety disorders.

Pediatrics is different from adult medicine in many ways because a child is different physiologically from an adult. As a result, congenital defects, genetic variance, and development‐related disorders are of great concern to pediatricians. Children are not simply miniature adults. A child's immature and developing physiology must be taken into account when considering symptoms, prescribing medications, and diagnosing illnesses. In particular, pediatric physiology greatly impacts the pharmacokinetic properties (absorption, distribution, metabolism, and elimination) of medications in developing children. Certain legal issues further complicate the delivery of pediatric health care since children are minors and, in most jurisdictions, cannot make decisions for themselves. Thus, issues related to guardianship, privacy, legal responsibility and informed consent need to be considered in every pediatric procedure, although more recent studies indicate that children and teens are capable of making their own health decisions at the age of 12 or 13.^4^

For the past few decades, advances in biomedical research have significantly improved the lives and health of infants, children, and adolescents. In fact, Cheng et al., through an open‐ended survey of pediatric professionals, identified seven great achievements in pediatric research that have occurred in the past 4 decades: preventing infectious diseases with life‐saving immunizations, reducing sudden infant death with the Back to Sleep campaign, finding a cure for acute lymphoblastic leukemia, improving the respiratory function of premature infants with surfactant therapy, preventing HIV transmission from mother to infant, increasing the life expectancy for children with sickle cell anemia and cystic fibrosis, and saving lives with car seats and seat belts.^5^ Furthermore, the same group predicted the next seven great achievements in pediatric medicine, which include more effective pediatric immunizations to prevent emerging and persistent diseases, promising immunotherapy to treat pediatric cancers, novel genomic discoveries to predict, prevent, and treat pediatric diseases, extensive lifelong data to identify fetal and childhood origins of adult health and disease to allow for early interventions, better understanding of the social‐environmental influences on biology and health to improve individual and population health, quality improvements to establish safe and efficient systems of care worldwide, and the implementation and dissemination of pediatric research to reduce poverty on a global scale.^6^

While it is difficult to predict any scientific discovery, since these are usually driven by seminal insights from unexpected directions, it is undoubtedly true that we are at an extraordinary time of innovative technologies. We believe we are on the edge of exciting discoveries and initiatives to improve the lives of children and the adults they will become. In fact, the biomedical sciences are exploding with new knowledge that is continuing to expand the research horizons. Innovative technologies and interdisciplinary approaches in areas of genomics, proteomics, transcriptomics, pharmacogenomics, epigenetics, metabolomics, nanosystems, and bioinformatics have allowed us to conquer numerous scientific challenges more efficiently than ever before, leading to faster translation of observations from bench to bedside and vice versa. It is therefore imperative that the new discoveries and advances in all aspects of basic, translational, and clinical pediatric medicine be rapidly disseminated to the professionals involved in pediatric care.

*Pediatric Discovery* will serve as a timely and valuable platform for publishing and propagating the important and state‐of‐the‐art discoveries that may fundamentally affect child and adolescent health. In this era of scientific abundance and the availability of a vast array of information and technology, our journal is positioned to strive toward more effective communication with and education of the pediatric community regarding the practical meaning of the important scientific milestones achieved in medicine in general, and pediatric medicine in particular.

The ultimate goal of the journal is to be a voice for basic, translational, and clinical researchers and professionals to communicate their innovative observations, which may lead to future interventions and the prevention of pediatric disorders. Through our partnership with John Wiley & Sons, Inc., we will make the submission and review process seamless by creating a central editorial office for *Pediatric Discovery*, which will receive all manuscripts from around the world. The paperless review process will help decrease the turn‐around time without compromising the quality or even‐handedness of the review process. We will encourage pediatric scientists and professionals from all disciplines in pediatric medicine to submit their best and/or most promising work to *Pediatric Discovery* to raise the standing of the journal. We believe these efforts will set the stage for progress as pediatric science evolves and will ensure that our journal is at the forefront of pediatric research.

A key aspect of *Pediatric Discovery* is its commitment to evidence‐based, as we recognize the critical role this plays in improving patient outcomes and the overall quality of care. The journal will encourage the submission of systematic reviews and clinical practice guidelines, which provide a solid foundation for evidence‐based practice by synthesizing the best available research and expert consensus. Furthermore, *Pediatric Discovery* will strive to contribute to the development of high‐quality guidelines and promote the use of rigorous methodologies in their creation, ensuring the highest standard of care for pediatric patients.

Although overseen by the National Clinical Research Center for Child Health and Disorders of China, and the Children's Hospital of Chongqing Medical University, *Pediatric Discovery* serves the global academic and professional communities across all pediatric disciplines. We strive to meet the expectations of the pediatric community and depend on their assistance and support to overcome challenges and ultimately establish *Pediatric Discovery* as a leading journal in pediatric medicine.

Last but not least, as the founding Editors‐in‐Chief, we are honored and grateful for the trust placed in us and the opportunity to represent *Pediatric Discovery*.

## Data Availability

Data sharing not applicable to this article as no datasets were generated or analyzed during the current study.
